# An Individual Prosthesis Control Method with Human Subjective Choices

**DOI:** 10.3390/biomimetics9020077

**Published:** 2024-01-27

**Authors:** Lei Sun, Hongxu Ma, Honglei An, Qing Wei

**Affiliations:** College of Intelligence Science and Technology, National University of Defense Technology, Changsha 410073, China; sunlei17a@nudt.edu.cn (L.S.);

**Keywords:** lower limb prosthesis, gait trajectory, planning, individual algorithm, subjective choices

## Abstract

An intelligent lower-limb prosthesis can provide walking support and convenience for lower-limb amputees. Trajectory planning of prosthesis joints plays an important role in the intelligent prosthetic control system, which directly determines the performance and helps improve comfort when wearing the prosthesis. Due to the differences in physiology and walking habits, humans have their own walking mode that requires the prosthesis to consider the individual’s demands when planning the prosthesis joint trajectories. The human is an integral part of the control loop, whose subjective feeling is important feedback information, as humans can evaluate many indicators that are difficult to quantify and model. In this study, trajectories were built using the phase variable method by normalizing the gait curve to a unified range. The deviations between the optimal trajectory and current were represented using Fourier series expansion. A gait dataset that contains multi-subject kinematics data is used in the experiments to prove the feasibility and effectiveness of this method. In the experiments, we optimized the subjects’ gait trajectories from an average to an individual gait trajectory. By using the individual trajectory planning algorithm, the average gait trajectory can be effectively optimized into a personalized trajectory, which is beneficial for improving walking comfort and safety and bringing the prosthesis closer to intelligence.

## 1. Introduction

As a commonly used auxiliary device, a lower limb knee–ankle prosthesis can effectively assist knee amputees to complete basic daily activities. An intelligent knee–ankle prosthesis is an important application in the field of human–robot interaction (HRI), which has a close connection with humans. In [1], the authors raised a three-layer control structure in which the joint trajectory planning belongs to the perceptual layer and provides control instructions for the lower layer control, where the model is normally used. Because people have their own walking modes, it is necessary to provide individual trajectories to satisfy individual requirements, thus improving the comfort and stability of wearing intelligent prostheses.

At present, the adjustment of lower limb prosthetic parameters mainly relies on manual adjustment by prosthetic clinicians or professional technicians. This method has some disadvantages: Firstly, it is reliant on manual operation. Prosthetic clinicians need to have rich experience and skills to accurately evaluate and adjust the adjustment parameters according to the specific conditions of the patients. In addition, manual adjustment requires a lot of time and effort because each patient’s physical condition is different and needs personalized adjustment. Furthermore, the user’s judgment of the best mode is inaccurate and cannot specify the most comfortable parameters. There may be some deviation between the user experience and the adjustment described by the user, which may not correspond to the optimal comfort level. In order to overcome these disadvantages, some institutions have begun to adopt automatic adjustment technology, which can achieve rapid and accurate parameter adjustment through sensors and intelligent control algorithms. This method can greatly shorten the adjustment time, improve parameter precision, reduce patient dependency, and improve the use effect of prostheses. However, at present, this automatic adjustment technology is still in the research and development stage and has not yet been fully popularized.

An individual prosthesis is controlled by a customized controller that considers the wearer’s motion habits and features. The authors in [2,3,4,5,6,7] proved that walking gait can be affected by sex, age, height, body weight, body mass index (BMI), and the walking habits of different subjects. It is vitally important to study the individual gait trajectory generation of prostheses to improve comfort levels. However, more attention should be paid to individual algorithm research.

Recent research on individual gait analysis has primarily focused on medicine and human physiology. A commonly used method is to build a regression model based on the gait data. In Ref. [8], a method for predicting lower limb sagittal kinematics was proposed, using multiple regression models based on walking speed, gender, age, and BMI as predictors. In [9], age, height, and weight were input into the regression model as the influencing factors. The data were then stratified by sex, adjusted to a common height using a pendulum model, and a simple regression for each parameter was performed. These studies have statistical significance in objective conditions, but they cannot solve the individual problem of subjective factors that are determined by walking habits.

Based on the virtual constraint control method, [10] proposed a gait kinematic modelling method that considers walking state differences. In 2020, the team implemented personalized method modelling based on this algorithm by modifying the benchmark model in [11]. In addition, [12] used a Gaussian process-enhanced Fourier series to model individual continuous joint kinematics, and [13] proposed an individual method for estimating walking speed. The subjects walked a few steps at a walking speed chosen by themselves. According to the data, scale factors representing the gait characteristics of the experimenter were calculated and used to achieve personalization in the machine learning algorithm to estimate walking speed. The proposed individual methods can reduce the computational burden to satisfy the real-time application scenario. These individual methods require the subject to walk on level ground for a certain distance to then calculate deviations between the average gait model and the actual walking. They realized individuation by adding the deviation to the average model. This method uses only one experiment to adjust the model parameters. However, the deviation changed when the motion conditions changed. In addition, for amputees, the kinematic model cannot be modified by walking naturally on flat ground.

In 2019, [14] presented a personalized gait optimization framework for lower-body exoskeletons. Users can provide preferences for a given pairwise preference between trials and suggest improvements. This provided an innovative research idea for individual gait optimization which was applied to the exoskeleton. This study refers to this method of preference selection and provides an individual trajectory optimization method that is more suitable for prosthetic control systems.

Considering the advantages and disadvantages of previous studies, the following aspects were considered:

1. This study introduces subjective human choices into prosthesis trajectory planning. Human subjective choice is defined as choosing the option that is more subjectively comfortable through human independent judgment under comparison, rather than optimization based on sensor data or calculated indicators under a fixed control paradigm, and taking human preference as important feedback.
2. This study focuses on planning the individual trajectory by reconstructing the curve by optimizing the positions of the key points instead of the gait features such as the step length and width as shown in [14]. It can be easily modelled according to the gait phase variable and does not require the transfer of gait features into joint angles.
3. We built a deviation function under different actions (the parameters needed to be optimized) through Fourier series expansion and discovering the coefficients required to evaluate the performance, which has an advantage over the method that estimates every utility function of the actions that can realize trajectory optimization with fewer iteration steps.

The remainder of this paper is organized as follows. Section 2 introduces the dynamic lower-limb prosthesis prototype and the virtual constraint method used in the control system. Section 3 describes the individual algorithm in detail from three parts: dataset and data preprocessing, gait curve reconstruction, and individual algorithms with subjective choices. Section 4 presents the experiments and results. Section 5 and Section 6 present the discussion and conclusions, respectively. The prosthetic knee joint angle was taken as an example to verify the method used in this study.

## 2. Dynamic Prosthesis and Virtual Constraint Method

### 2.1. Dynamic Knee–Ankle Prothesis

**Figure 1:**
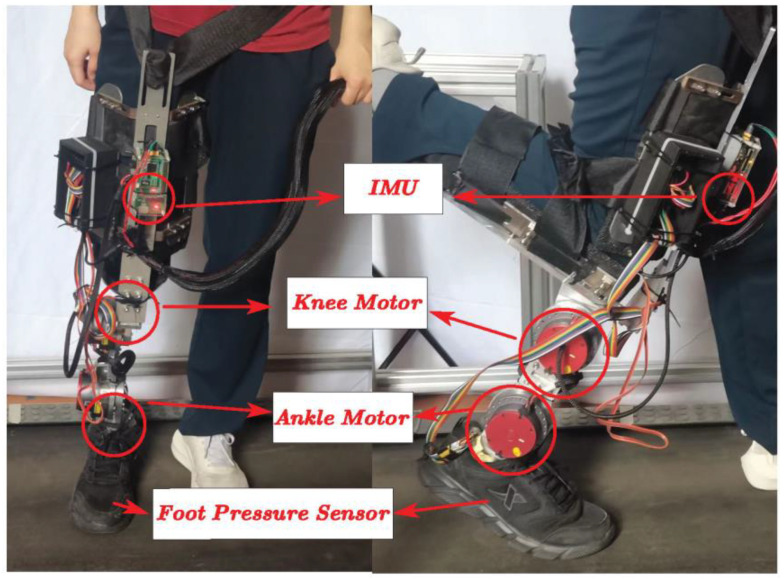
The dynamic prosthesis prototype.

In this study, a dynamic lower-limb prosthesis built in the laboratory was used, as shown in Figure 1. Two motors were used at the knee and ankle joints to provide active torque and real-time feedback of the joint position and angular speed. The two motors replace the function of human joints and are connected in series by a rigid connecting rod whose length can be adjusted to suit different modes of wear.

To facilitate the researchers to test the performance of the prosthesis, a shank lower frame was designed to fix the shank of the subject at a certain angle to the back side. The upper part of the prosthesis is bound to the human thigh through a rigid mechanism to ensure that the relative sliding or rotation between the prosthesis and the human leg is as small as possible during walking. The lower frame can be removed when it is worn by amputees. A prosthetic prototype can be used to verify the performance of the mechanism design and control algorithm. In addition, inertial measurement units (IMUs) and foot pressure sensors are installed for human gait data acquisition, human gait phase recognition, and subsequent data processing.

### 2.2. Virtual Constraint Control

In this study, a virtual constraint control method based on the human residual thigh angle was used. The phase variable reflects the phase of the gait cycle in which the current moment is and can be expressed as φ∈{R|0≤φ≤1,φ>0}, which monotonically increases over a complete period. The commonly used phase variable is calculated using the thigh angle and angular velocity ([15,16]), but the angular velocity is easily disturbed by noise. Therefore, the residual thigh angle and its integral are used to obtain the phase variable φ, which reflects the subjective initiative of the user and has a better aperiodic property. The thigh angle $q_{t}$ is defined as the angle between the thigh and vertical direction. The integral of $q_{t}$ can be expressed as $Q_{t}=\int_{0}^{t}q_{t}(\tau )d\tau$. Then, the phase variable φ is expressed as:

(1) $$\varphi \left(t\right)=atan2(\left(Q_{t}\left(t\right)+\Gamma \right)z,\;q_{t}\left(t\right)+\gamma )$$

where scaling factor z, thigh angle offset γ, and thigh angle integral offset Γ are defined as follows:

(2) $$z=\frac{{|q}_{t,max}-q_{t,min}|}{\left|Q_{t,max}-Q_{t,min}\right|}$$

(3) $$\gamma =-\frac{q_{t,max}+q_{t,min}}{2}$$

(4) $$\Gamma =-\frac{Q_{t,max}+Q_{t,min}}{2}$$

**Figure 2:**
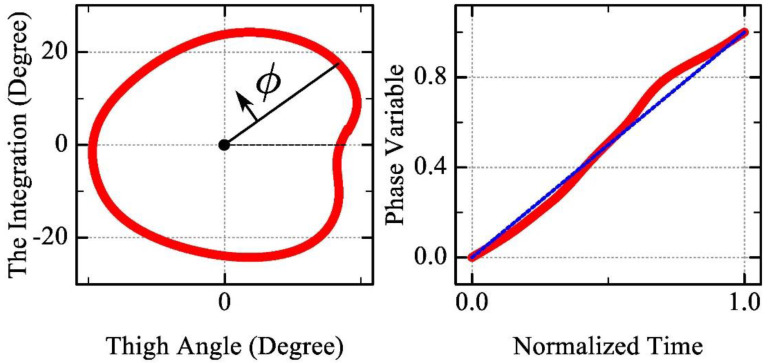
The calculation result of the phase variable in one gait cycle. The red line is the phase variable calculation results and the blue line is the reference line with a slope of 1.

These parameters are used to adjust the shape and position of the track and improve linearity. The integral was reset at each step to eliminate accumulated errors. Figure 2 gives the calculation result of the phase variable in one gait cycle.

Using the virtual constraint control method, the periodic continuous gait curve can be obtained by the phase variable, which greatly simplifies the difficulty of tuning the impedance control parameters of the finite-state machine. Besides, using phase variable instead of the time variable has the advantage of human dominance, the prosthetic leg moves according to the human’s intention. It is important to design a reasonable gait trajectory that can be adapted to individual walking characteristics.

Based on this virtual constraint control method, “time-based control” is transformed into “phase-based control”. The detection of the gait stage is not based on absolute time but is determined by the state of the thigh angle, which can better cope with the influence of walking speed change or non-periodic movement. Even if the gait frequency changes, the phase position of the phase variable calculated by this method will not change during the gait cycle, corresponding to the same position of the gait trajectory, which can realize the integrated and unified control of the knee and ankle joints during the whole gait cycle.

## 3. Methods

**Figure 3:**
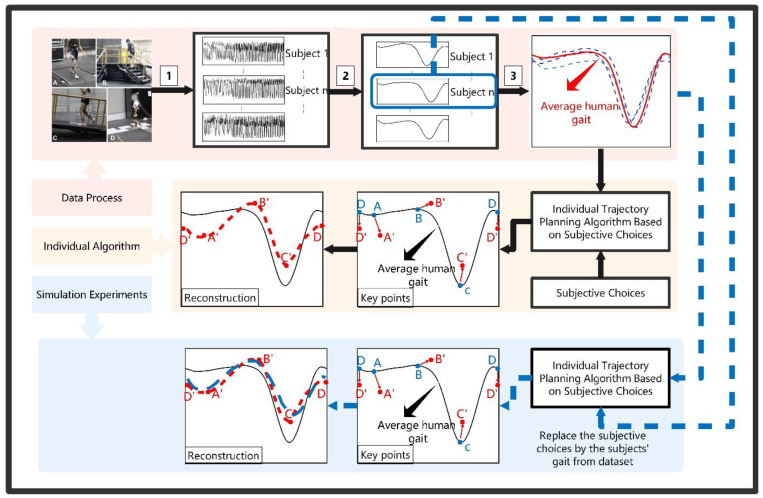
The main concept of this paper, comprising 3 parts: data processing, individual algorithm, and simulation experiment. The number 1–3 represents the data processing steps. Step 1 collects the raw data from sensors, step 2 divides the raw data into gait cycle by heel-strike point and step 3 calculate the average gait trajectory of all the subjects’ gait cycle.

Figure 3 presents the main concept of this study. It comprises 3 parts: data processing, individual algorithms, and simulation experiments. In the data processing part, an open-source gait dataset is leveraged to obtain the subjects’ actual individual gait, human average gait, and other data support. The dataset and the data processing method are introduced below. Part 2 uses the results of Step 3 and the subjects’ subjective choices as the inputs of the individual trajectory algorithm. By optimizing the position of the key points of the curve, a reconstructed individual curve is obtained. The flow of the simulation experiments is shown in Section 3. To verify the effectiveness of the algorithm, we designed simulation experiments before physical use. One difficulty is that it is hard to obtain a human’s subjective choices in the simulation stage, so we used the subjects’ actual average gait as a reference. It is intuitive to observe the results from the comparison of the three gait curves: human average gait (HAG), the subject’s actual gait, and the optimized individual gait (IG) using the algorithm.

### 3.1. Dataset and Data Preprocessing

This study used the dataset provided in [17], which greatly supports our research. This dataset contains 3-dimensional biomechanics and wearable sensor data from 19 able-bodied adults. In this article, we focused on the lever-ground walking mode. In this mode, subjects walked on a treadmill with speeds ranging from 0.5 to 1.85 m/s in 0.05 m/s increments, which includes the walking speeds of common daily life activities. We defined the knee joint angle and ankle joint angle as $q_{ki}$ and $q_{ai}$ with i=1,2,⋯,19, which represents the different subjects in the dataset. The single-cycle gait of each subject starts from the plantar pressure from one heel-strike point to the next. As shown in Part 1 in Figure 3, Step 1 extracts every subject’s joint angle at the same speed. Step 2 divides every gait step by the heel-strike point and then averages all the steps to obtain the individual gait of each subject in a gait cycle. The mean value of the result of Step 2 was taken as the average human gait of this dataset.

**Figure 4:**
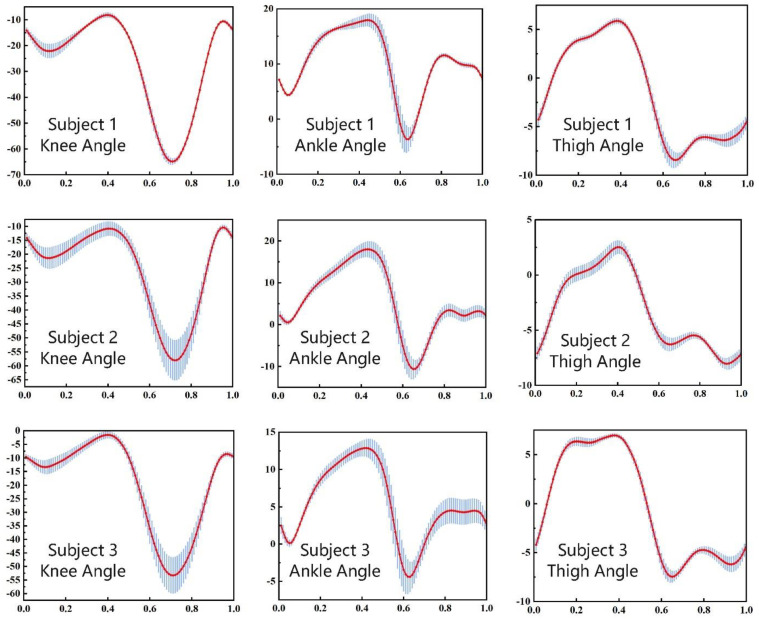
Knee angles, ankle angles, and thigh angles of 3 subjects. The horizontal axis represents the phase variable of one gait cycle that has no units, the longitudinal axis is the joint angle in degrees. The red line is the mean value and the blue line is the interval range.

Individual gait characteristics are highly dependent on the subjects’ walking habits and other body conditions, which contain many factors that are complex to model. For a definite subject, the gait curves of each cycle may differ. As shown in Figure 4, three subjects had differences in walking. During one continuous experiment with the same walking speed, the knee angle, ankle angle, and thigh angle fluctuated around the average value and followed a certain rule. This gives us a chance to redescribe the curve according to the law.

### 3.2. Gait Curve Reconstruction

**Figure 5:**
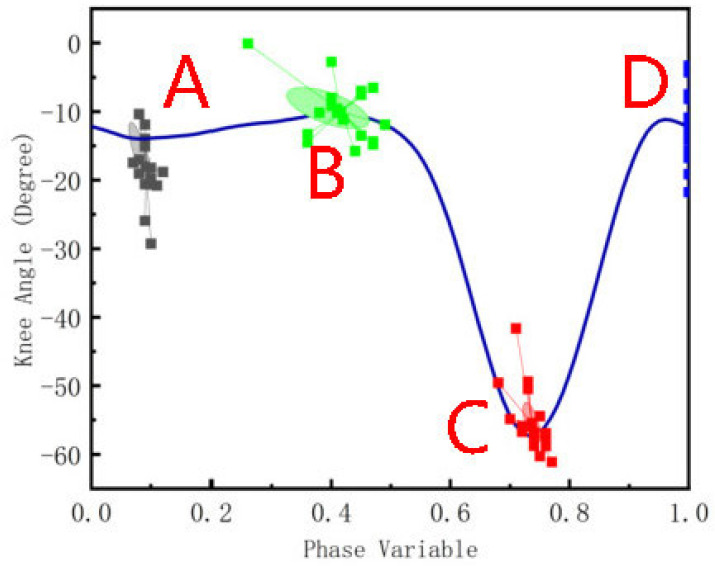
Four key points of the knee angle. A is the first minimum point of the curve, B is the first maximum point, C is defined as the minima point during the cycle, and D is the point when the phase variable is 0/1.

By analyzing the numerical differences of knee angles in one gait cycle of the 19 subjects in the dataset, including the subjects’ sex, height, weight, and the coordinates of the four chosen key points shown in Figure 5, it can be seen that different subjects have certain differences in the coordinate values of the key points, whether from the aspect of gait phase dimension or knee angle. The maximum difference in the joint angles is approximately 20 degrees, while the maximum difference in the gait phase was 0.23. The joint curves can be reshaped by adjusting the positions of the key points to better fit the individual differences among the subjects. The coordinates of the key points determine the shape of the curves from one aspect, reflecting actual walking; they reflect the step length, the height of the knee, and other important characteristic features.

We chose four key points to reconstruct the gait curve, as shown in Figure 5. The 4 key points largely determine the curve shapes, which are the minimal and maximal points. Although a higher number of key points means that the gait curve reconstruction is more accurate, it takes a longer time to adjust the position of each key point. It is important for prosthetic users to obtain personalized gait trajectory planning results with fewer steps. Thereafter, the horizontal or vertical coordinate stretches according to the new key points to maintain the basic shape.

### 3.3. Individual Trajectory Optimization with Subjective Choices

In this section, an individual trajectory optimization method is proposed to optimize user comfort based on subjective human choices. People’s feelings are the most intuitive and accurate evaluation method for prosthetic products, and it is difficult to describe the feelings using specific and quantitative equations in many cases. It is necessary to introduce human subjective evaluation as the criterion of system optimization for this type of human–robot interaction system.

#### 3.3.1. Fourier Series Expression of the Deviation Function

$K=\left\{A,B,C,\cdots \right\}$ is defined as the set of key points, and the coordinate $c_{k}$ needed to be adjusted, where c∈{x,y} represents the horizontal or vertical coordinate and k∈K. We assume that every choice of $c_{k}$ leads to a definite deviation $D(c_{k})$ that can be regarded as the uncomfortable feelings of users when they make a subjective choice. The deviation function is expressed as a Fourier series expansion in n order:

(5) $$D\left(c_{k}\right)=\frac{a_{0}}{2}+\sum_{i=1}^{n}\left(a_{i}\cos\left(ic_{k}\right)+b_{i}\sin\left(ic_{k}\right)\right)$$

(6) $$a_{i}=\frac{1}{\pi }\int_{-\pi }^{\pi }\cos\left(ic_{k}\right)dc_{k}$$

(7) $$b_{i}=\frac{1}{\pi }\int_{-\pi }^{\pi }\sin\left(ic_{k}\right)dc_{k}$$

where we define $X={\left[a_{0},a_{1},\cdots ,a_{n},b_{1},\cdots ,b_{n}\right]}^{T}$ as the set of Fourier series variables.

#### 3.3.2. Posterior Probability Calculation

We define a Gaussian process model of X:

(8) $$\;\;P\left(X\right)=\frac{1}{{\left(2\pi \right)}^{\frac{N}{2}}{\left|\Omega \right|}^{\frac{1}{2}}}e^{\frac{1}{2}{\left(X-\mu \right)}^{T}\Omega^{-1}{\left(X-\mu \right)}^{T}}$$

where $N=2n+1,\;\mu \subseteq R^{N\times 1}$ and $\Omega \subseteq R^{N\times N}$ represent the mean value and the covariance matrix, respectively. We are interested in the posterior probability P(X|Sc) where Sc (Subjective Choice) represents the preference set after every subjective choice. According to Bayes’ theorem, we obtain:

(9) $$\;P\left(X|Sc\right)=\frac{P\left(Sc|X\right)P\left(X\right)}{P(Sc)}$$

where $P\left(Sc\right)$ is a constant commonly used for normalization. $P\left(X\right)$ is the prior probability defined above. Substituting the result of subjective choices into probability P(Sc|X) yields:

(10) $$P\left(Sc|X\right)=P(D\left(c_{k1}\right)>D(c_{k2})|X)$$

where the inequality $D\left(c_{k1}\right)>D(c_{k2})$ indicates that the subject chooses a joint trajectory with $c_{k2}$. The cumulative distribution function is used to express the comparison of the two deviations within the two choices [14]:

(11) $$\begin{array}{cc} P\left(Sc|X\right) & =\prod_{a,b}\int_{c_{ka}}^{c_{kb}}D(x)dx\\ & =\sum_{a,b}\Phi [D\left(c_{ka}\right)-D(c_{kb})] \end{array}$$

where Φ(·) is the standard normal cumulative distribution function.

#### 3.3.3. Maximum A Posteriori Estimation

The maximum a posteriori estimation (MAP) method is used to update the value of X. The target of MAP is:

(12) $$\begin{array}{cc} {\hat{X}}_{MAP} & =\arg\underset{X}{\max}lnP(Sc|X)P(X)\\ & =\sum_{a,b}\Phi [D\left(c_{ka}\right)-D(c_{kb})]+lnP(X)\\ & =\sum_{a,b}\Phi [D\left(c_{ka}\right)-D(c_{kb})]+\frac{1}{2}{\left(X-\mu \right)}^{T}\Omega^{-1}{\left(X-\mu \right)}^{T} \end{array}$$

where ${\hat{X}}_{MAP}$ represents the estimation of X. Substituting $\hat{X}$ into Equation (5), it is possible to obtain the optimal ${\hat{c}}_{k}$ value that minimizes D. $\sum_{a,b}\Phi [D\left(c_{ka}\right)-D(c_{kb})]$ is the sum of all subjective choices that are recorded in set Sc in the completed and current iterations.

## 4. Experiments and Results

### 4.1. The Optimization Algorithm Pseudo-Code

In this section, we provide a detailed algorithm pseudo-code in Algorithm 1.
**Algorithm 1 Framework of individual gait optimization algorithm**1: **procedure** INDIVIDUAL GAIT OPTIMIZATION AlGORITHM 2:   Select a key point position $c_{k}$ to be optimized. 3:   Initialize the Fourier Series parameters X. 4:   Initialized μ and Ω. 5:   **for** each t=1:T **do**
6:      **for** each i=1:k **do**
7:        Sample k values of X. 8:        Choose the value of X with maximum probability. 9:      **end for**
10:       Select 2 actions of $c_{k}$ from the optional value within the predefined range by random. 11:       Subjective choice between the 2 actions under $c_{k}$ and refresh the choice matrix. 12:       Use the MAP algorithm to update the value of X and get the optimal $c_{k}$. 13:    **end for**
14: **end procedure**

In the simulation experiment, we have explained the implementation as below:

1. To be closer to real situations where people make decisions on which option they prefer, the real deviation is added by Gaussian noise with a 30% error band.
2. The number of actions to be selected is determined. In this study, the number of actions to be selected can be effectively reduced by directly fitting the parameters of the deviation function, and the gait curve can be optimized by fitting the deviation function with fewer subjective selections. Considering the accuracy and efficiency comprehensively, five actions were selected for each parameter to be optimized.
3. By comparing the individual average gait curve of the dataset, the subjective choices of humans in the simulation experiments were replaced. In the simulation experiments of the algorithm verification, it was necessary to obtain subjective human choices as the input. Therefore, we used the individual average gait as a standard value. By calculating the difference between the positions of the key points, we substituted the subjective feelings to make a choice.

### 4.2. The Optimization Algorithm Verification

**Figure 6:**
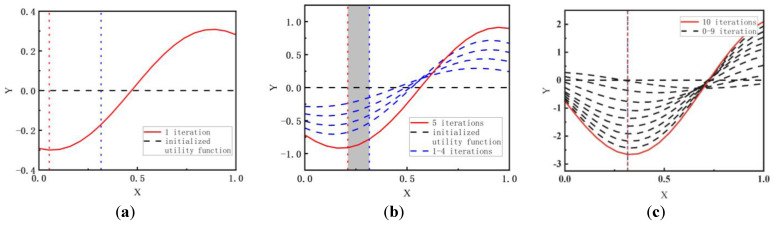
Knee angles, ankle angles, and thigh angles of 3 subjects. (**a**) 1 iteration; (**b**) 5 iterations; (**c**) 10 iterations. The red line is the latest iteration result and the shaded areas between vertical red and blue lines are the errors of the estimation results.

To confirm the validity of the individual trajectory optimization method, we first verified the method using a one-dimension deviation curve, where the domain of definition X is (0,1) and the dependent variable is Y. The results are shown in Figure 6. Figure 6a shows the initialization of the deviation function (the variables were set to 0) and the result after one iteration. Figure 6b and Figure 6c show the simulation results after five and ten iterations, respectively. The red dotted line vertical to the X-axis indicates the minimum point in the current iteration, and the blue dotted line vertical to the X-axis is the actual minimum point. In this experiment, the action pairs of each iteration were selected randomly, which may lead to a different convergence process. As the number of iterations increases, the optimum value gradually approaches the desired area.

**Figure 7:**
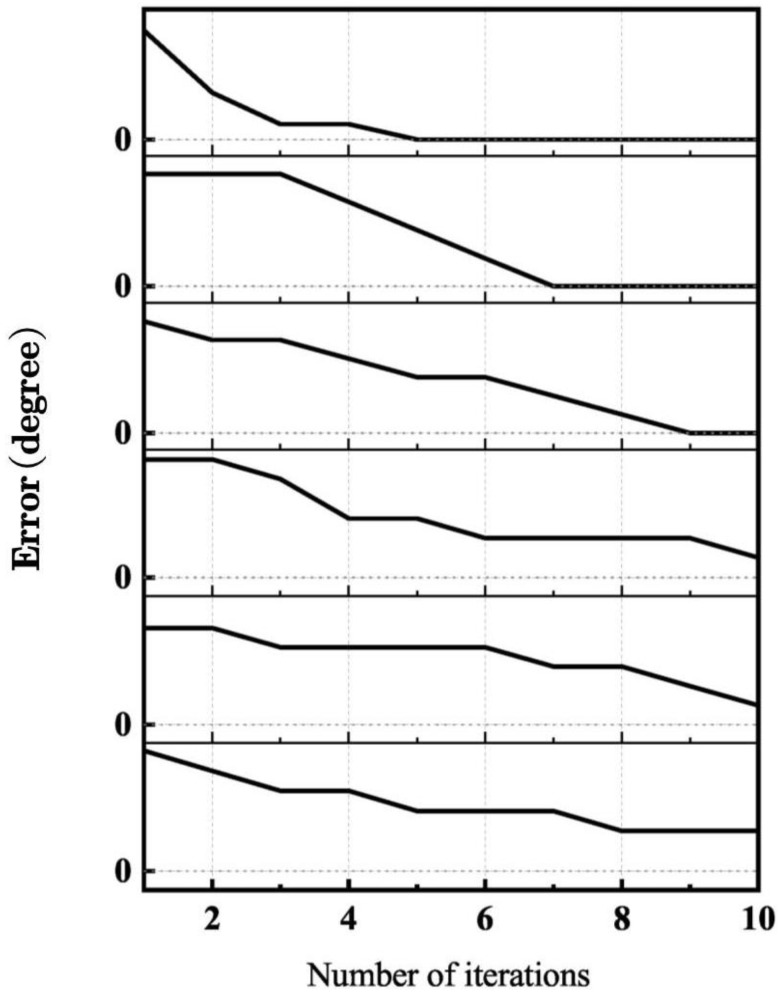
The error of each iteration in 6 experiments. The horizontal axis represents the number of iterations and the longitudinal axis is the optimization results’ errors.

Figure 7 shows the error of every iteration relative to the minimum point in six repeated experiments with 10 iterations. The results of the first three experiments show that the errors converge to 0 after 10 iterations. The latter three experiments produced errors that decreased but did not reach 0, as the random-action selection method affected the process of convergence to a minimum deviation point. When an effective action is selected, the convergence is faster; otherwise, the convergence rate is affected. This can be solved by increasing the number of iterations or by developing a method for effective action selection.

### 4.3. Dataset-Based Gait Simulation

In this part, the gait dataset mentioned above is leveraged to verify the individual trajectory optimization method used for a specific human gait. Consider the horizontal coordinates of point C as an example to display the results. We used the gait data at a speed of 0.5 m/s, and took the average of all subjects’ single gait as a standard instead of human subjective feeling. In the simulation experiment, human choice could not be obtained by the physical wearable experience. The Fourier order n of the deviation function was set to 5. The optional value range was determined by analyzing the dataset to determine an approximate range to cover most people’s walking habits.

**Figure 8:**
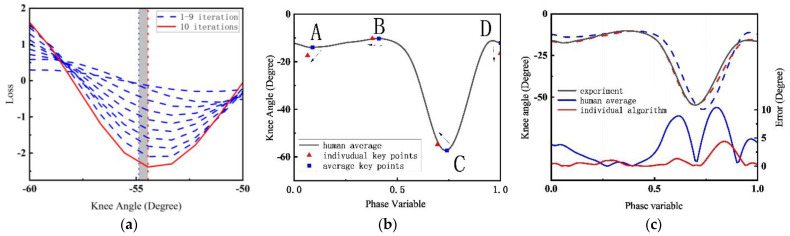
The gait curve optimization process. (**a**) 10 iterations of the horizontal coordinate of key point C. (**b**) Determine the key point from the average to individual position. (**c**) Reconstruct the curve and show the errors of average gait and individual gait separately.

Figure 8 shows the process of the algorithm for a subject’s knee joint angle. Figure 8a converges to an optimal value to adjust the positions of all key points. The red line is the results of the 10 iteration, and the blue lines are the 1-9 iterations. Intuitive adjustments of the key points are shown in Figure 8b,c, which reconstructs the curve using the quintic spline method. The black line is the result of this algorithm, the red line is the average knee joint angle calculated by the data of one of the subjects at 0.5 m/s, and the blue line is the human average knee joint angle calculated by all subjects’ data.

Table 1 lists the results of the experiments for all 19 subjects. Δ represents the difference between the optimal and average coordinate values. Three indexes are calculated to explain the effect of the optimization algorithm with the same unit of degree. The Euclidean Distance (*ED*) is a commonly used index, which is a point-based method to describe the similarity of two curves and is defined as

(13) $$ED=\frac{1}{100}\sqrt{\sum_{i=1}^{100}\left[{\left(x_{i}^{HAG}-x_{i}^{IG}\right)}^{2}+{\left(y_{i}^{HAG}-y_{i}^{IG}\right)}^{2}\right]}$$

The Fréchet distance (Fre) and Hausdorff distance (Haus) are shape-based distance descriptions defined as

(14) $$Fre=\underset{\alpha ,\beta }{\inf}\underset{\varphi \in [0,1]}{\max}d(x_{i}^{HAG}\left(\alpha \left(\varphi \right)\right),\;x_{i}^{IG}\left(\beta \left(\varphi \right)\right))$$

(15) $$Haus=\max(h\left(x_{i}^{HAG},\;x_{i}^{IG}\right),\;h(x_{i}^{IG},\;x_{i}^{HAG}))$$

where d represents the distance between the two sample points and $h\left(A,B\right)=\underset{a\in A}{\max}\underset{b\in B}{\min}∥b-a∥$. As shown in Table 1, the individual trajectory optimization algorithm has a better fitness than the average trajectory, not only from the point-based distance but also from the shape-based distance.

The algorithm effectively makes the joint trajectory more consistent with the individual’s walking habits, which cannot be calculated by collecting the daily gait data of prosthetic users. Through this subjective judgment method, information about people’s feelings can be collected and used. During the experiment, with an increase in the number of iterations, it converged to the desired optimal result. However, experiments involving humans, especially people with disabilities, are not always suitable for frequent repetition. Ten iterations were chosen here, and within this number, it can converge to the allowable range of a small error.

### 4.4. Results Analysis

**Figure 9:**
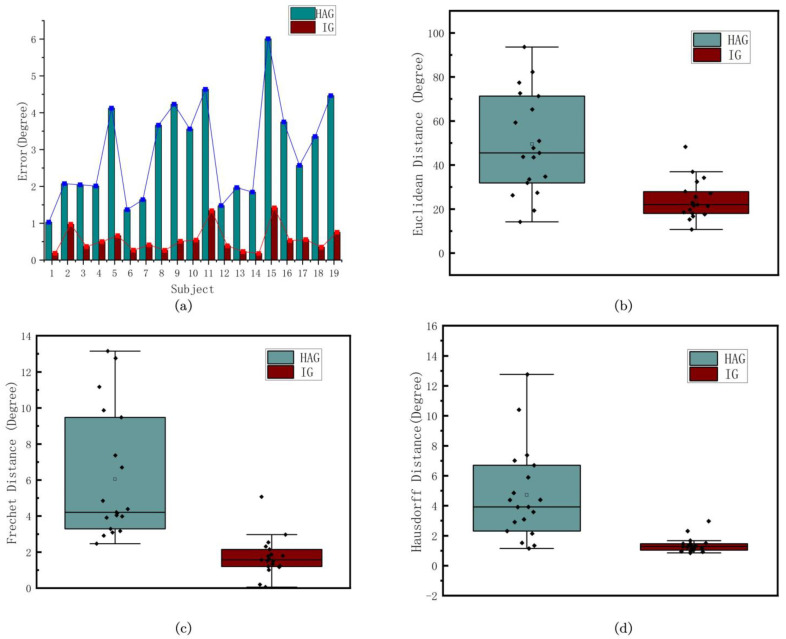
(**a**) shows the errors of the coordinates of the 4 key points of HAG and IG, respectively, where the units are degree or phase (depending on the coordinate type). (**b**–**d**) are the 3 indexes to describe the optimization results, which are the Euclidean distance, Fréchet distance, and Hausdorff distance, the units of which are degree.

Table 1 gives the detailed optimization results of all 19 subjects, including eight key points and three indexes. Figure 9a shows the coordinate errors of the key points in the 19 experiments. Figure 9b,c represents the box plots of the three indexes. It is obvious that the IG results performed better than the HAG results, with lower errors in the coordinates of the key points in general. The mean Euclidean distances of HAG and IG are 49.49 and 24.17, the mean Fréchet distances are 6.05 and 7.17, and the mean Hausdorff distances are 4.72 and 1.38, respectively. We can see from the indices that after gait trajectory optimization, the gait curve is closer to the real individual curve from the distance and shape aspects.

Most of the subjects’ gait curves could be optimized to be close to the actual curve, and the similarity was significantly improved. For subjects whose key points are approaching the average gait, the optimization space is limited and the coordinate errors may be larger than the original curve error. However, overall, the curve distance and shape similarity improved.

## 5. Discussion

### 5.1. Advantages of Individual Gait Trajectory Optimization

In this study, we proposed a gait trajectory optimization method based on subjective choices. In the human–robot interaction control system, the human is a key link in the loop as this method uses the subjective feedback of humans. It introduces the hard-to-quantify comfort index into trajectory optimization by paired preference selection, which is different from the traditional methods that only use objective indices calculated from sensor data. The proposed method is an off-line optimal process that is uniformly optimized after a walk and then used for the next walk after adjusting the trajectories.

Four key points were selected to reconstruct the gait curve, and we chose points with physical significance or the peak points of the curve. When reconstructing the curve, the basic shape is maintained to ensure the physiological characteristics of the joints during walking. For intelligent prosthesis researchers, the direct optimization of the gait curve can reduce the complexity of the calculation compared to gait feature optimization.

To be more accurate in the real situation of human selection, we made some improvements in the experiments. Human choice is actually a probabilistic model, so we used the form of a probability function to represent the choice of the subject, so as to make the simulation experiment more similar to the physical experiment and facilitate the debugging and verification of the algorithm.

The experiments and results demonstrate the effectiveness of the proposed algorithm. With limited step numbers, the optimal gait can be approached by choosing the preferred motion. Both the point-based similarity index and shape-based similarity index show that, compared with the human average gait trajectory, this method makes the trajectory more similar to the subject’s own personalized average model.

### 5.2. Future Works

At present, the comparison of each action adjusts only one parameter, which requires the subject to walk several times to complete the experiment. In future work, a method to improve the optimization efficiency is considered so that it can converge to the optimal result faster. The improvements may include (1) multiple groups of actions are selected at the same time in each iteration; (2) optimizing the method of action selection to reduce the frequency of the actions with a large expected loss; and (3) adding dimensions so that it can optimize two or more parameters simultaneously in one iteration.

In addition, physical experiments will be conducted in which the subject wears a knee–ankle prosthesis, and the average gait model will be modified using the algorithm to resemble the subject’s comfortable gait more closely. A combination of the subjective evaluation scale and objective index was used to evaluate comfort.

## 6. Conclusions

This paper proposes a method for optimizing the trajectory of trajectory optimization based on subjective human choices. We used a Fourier series to express uncomfortable feelings under different trajectories and selected four key points to reconstruct the gait curve. The subject chose a preferred trajectory within a pair of trajectories. The positions of the key points are modelled by Gaussian processes, and the MAP method is used to update the value. Thus, the optimal position was calculated for each iteration. A simulation experiment based on a standard gait dataset demonstrated the effectiveness of this method.

In the future, we plan to apply the individual trajectory optimization method to actual prostheses, improve the convergence speed of the algorithm, and apply the method to the optimization of multi-dimensional parameters.

## Figures and Tables

**Table 1:** Trajectory optimization results of 19 experiments. HAG and IG are key points error and gait trajectory similarity index, respectively.

	AB06		AB07		AB08		AB09		AB10		AB11		AB12		AB13	
	HAG	IG	HAG	IG	HAG	IG	HAG	IG	HAG	IG	HAG	IG	HAG	IG	HAG	IG
$\Delta x_{A}$	0	0	3	0.5	1	0.1	0	0	1	0.1	0	0.3	0	0.1	1	0
$\Delta y_{A}$	2.47	0.38	2.38	0.22	3.3	0.78	4.53	1.08	12.8	0.53	4.16	0.19	1.42	0.77	1.78	0.22
$\Delta x_{B}$	1	0.25	5	3	3	0.75	0	0	3	0.75	1	0.25	2	0.25	6	0.75
$\Delta y_{B}$	0.08	0.19	4.25	1.64	3.4	0.71	1.06	0.83	2.53	0.58	0.5	0.43	2.13	0.62	4.38	0.11
$\Delta x_{C}$	0	0	1	0.05	0	0.3	2	1.1	0	0.3	1	0.1	2	0.35	6	0
$\Delta y_{C}$	2.91	0.32	1.52	16.7	3.91	0.18	4.39	0.95	4.38	2.97	0.02	0.43	3.1	1	4.85	0.31
$\Delta y_{D}$	0.74	0.33	0.38	0.68	0.7	0.12	2.11	0.04	6.16	0.06	2.9	0.45	0.84	0.22	2.62	0.73
ED	14.2	10.7	31.9	18.0	43.7	22.0	34.7	34.2	59.3	27.2	19.4	16.7	33.5	27.9	77.4	22.8
Fre	2.91	1.51	3.29	1.78	3.91	1.48	4.39	1.17	11.2	2.97	4.16	1.60	3.09	1.57	4.85	1.2
Haus	2.91	1.39	1.52	1.67	3.91	1.34	4.39	1.16	4.38	2.97	1.15	1.35	3.09	1.28	4.85	0.85
	**AB15** **AB27**		**AB16** **AB28**		**AB18** **AB30**		**AB19**		**AB21**		**AB23**		**AB244**		**AB25**	
	**HAG**	**IG**	**HAG**	**IG**	**HAG**	**IG**	**HAG**	**IG**	**HAG**	**IG**	**HAG**	**IG**	**HAG**	**IG**	**HAG**	**IG**
$\Delta x_{A}$	0 2	0.4 0.8	1 1	0.2 0.5	4 0	0.9 0.6	0	0	1	0.3	2	0.1	5	1	1	0.5
$\Delta y_{A}$	2.23 4.35	0.22 0.2	2.62 4.21	1.27 0.06	13.1 9.48	6.67 1.87	2.47	0.62	0.57	0.2	1.01	0.14	14.9	8.40	6.07	0.38
$\Delta x_{B}$	3 2	0.5 0.5	7 5	1 1	1 5	1 0.5	2	0.75	6	0	4	0	5	0.75	2	0.5
$\Delta y_{B}$	3.13 5.66	0.62 0.97	1.81 4.73	0.48 0.64	5.15 3.58	0.05 0.53	0.98	0.48	3.17	0.12	0.05	0.36	10.4	0.28	7.37	1.06
$\Delta x_{C}$	3 1	0.30 1	3 1	0.75 0.35	2 1	0.65 0.50	2	0.25	2	0.55	4	0.35	0	0.15	1	0.4
$\Delta y_{C}$	12.8 3.92	1.64 0.73	6.70 5.89	0.11 0.04	2.50 3.41	0.66 0.57	2.31	0.54	1.48	0.39	0.45	0.11	2.46	0.37	5.01	0.35
$\Delta y_{D}$	5.52 1.07	0.41 0.24	3.79 2.64	0.53 0.20	8.68 8.79	0.77 1.48	0.60	0.47	0.56	0.28	3.42	0.37	9.34	0.37	4.81	1.05
ED	82.3 43.5	19.7 18.5	50.9 47.7	21.4 15.3	72.6 71.3	36.9 25.6	26.2	22.6	27.4	32.4	45.5	17.6	93.6	48.3	65.2	21.6
Fre	12.8 4.04	2.31 0.20	6.70 4.21	1.80 0.06	9.87 9.48	2.54 1.87	2.47	1.01	3.17	1.33	3.98	1.23	13.2	5.07	7.37	2.15
Haus	12.8 3.92	2.31 0.95	6.70 5.89	1.50 1.24	7.01 3.58	1.47 1.04	2.31	0.97	2.14	1.24	1.34	0.92	10.4	1.46	7.37	1.14

## Data Availability

The data that support the findings of this study are available at [15].
